# Antibacterial and Antifungal Activities of PMMAs Implanted Fluorine and/or Silver Ions by Plasma-Based Ion Implantation with Argon

**DOI:** 10.3390/ma13204525

**Published:** 2020-10-13

**Authors:** Keiichi Kagami, Yoko Abe, Yukari Shinonaga, Rie Imataki, Takako Nishimura, Kyoko Harada, Kenji Arita

**Affiliations:** 1 Department of Pediatric Dentistry, Graduate School of Dentistry, Osaka Dental University, 8-1, Kuzuhahanazono-cho, Hirakata-shi, Osaka 573-1121, Japan; kagami-k@cc.osaka-dent.ac.jp; 2 Department of Pediatric Dentistry, School of Dentistry, Osaka Dental University, 8-1, Kuzuhahanazono-cho, Hirakata-shi, Osaka 573-1121, Japan; ghfgj093@yahoo.co.jp (Y.S.); imataki-r@cc.osaka-dent.ac.jp (R.I.); nisimura@cc.osaka-dent.ac.jp (T.N.); kyoko-w@cc.osaka-dent.ac.jp (K.H.); arita-k@cc.osaka-dent.ac.jp (K.A.)

**Keywords:** argon, fluorine, silver, PMMA, plasma-based ion implantation, surface modification, antibacterial effect, antifungal effect

## Abstract

The purpose of this study was to examine the anti-oral microorganism effects of fluorine and/or silver ions implanted into acrylic resin (PMMA) using plasma-based ion implantation (PBII) with argon gas. The experimental PMMA specimens were implanted with F and Ag ions alone or simultaneously by the PBII method using Ar or Ar/F_2_ gases and Ag mesh. The surface characteristics were evaluated by X-ray photoelectron spectroscopy (XPS), contact angle measurements, and atomic force microscopy (AFM). Moreover, the antibacterial activity against *Streptococcus mutans* (*S. mutan*s) and the antifungal activity against *Candida albicans* (*C. albicans*) were examined by the adenosine-5’-triphosphate (ATP) emission luminescence method. XPS spectra of the modified specimens revealed peaks due to F in the Ar/F and the Ar/F+Ag groups, and due to Ag in the Ar+Ag and the Ar/F+Ag groups. The water contact angle increased significantly due to the implantation of Ar, F, and Ag. In the AFM observations, the surface roughness of the Ar/F and the Ar/F+Ag groups increased significantly by less than 5 nanometers. The presence of F and Ag was found to inhibit *S. mutans* growth in the Ar+Ag and the Ar/F+Ag groups. However, this method provided no significant antifungal activity against *C. albicans*.

## 1. Introduction

Poly (methyl methacrylate) (PMMA) is widely used for dental applications, such as removable appliances and orthodontic appliances and retainers [1,2]. The desirable attributes of PMMA include low cost, high biocompatibility, and easy handling; consequently, it is commonly used in dentistry [2]. Dental appliances made of PMMA have direct contact with oral tissues, and because the oral cavity contains a variety of microorganisms, PMMA based appliances in the mouth are immediately covered by salivary components and colonized by a multitude of microorganisms that may form biofilms [3]. Biofilm formation on dental devices may cause dental caries, periodontal diseases, or inflammation of oral soft tissue [4,5,6]. Dentures that are worn by elderly patients contribute to their oral health by maintenance and restoring the chewing function, and may help maintain general health, such as cognitive function [7]. Furthermore, Takeuchi et al. suggested that wearing dentures could partially moderate the increased risk of incident pneumonia associated with aspiration risk in older nursing home residents [8]. Nevertheless, it is difficult for elderly people and people with disabilities who do not have access to intensive care to keep their dentures clean because of functional deterioration or disability. Thus, dental plaque is a significant risk factor for opportunistic infections and aspiration pneumonia in the elderly and immunocompromised individuals [9,10]. Therefore, there is a need for a simple and economical approach to reduce the adhesion of biofilm and denture plaque to acrylic dentures. 

The two major types of methods for functionalizing dental materials are classified into chemical and physical modification methods. In chemical methods, material is mixed with appropriate antibacterial agents. In this approach, care must be taken to avoid disrupting any original properties of the body material and to control the amount of agent released from the material [11,12]. Plasma-based ion implantation (PBII) is a physical modification method that shows promise in the surface modification of three-dimensional materials [13]. In PBII, even a three-dimensional sample having a complex shape can be implanted ions into the surface with good compatibility and uniformity without beam scanning or special target manipulation. In our previous work, an approach to induce antimicrobial properties on a PMMA surface by modifying the material surface using the PBII method was proposed [14,15,16], and it was demonstrated that fluorine (F) and silver (Ag) ion implantation inhibited the growth of general bacteria, *Staphylococcus aureus* (*S. aureus*), on the PMMA surface [16]. However, how this method affects oral microorganisms was not verified.

Oral microbial communities are some of the most complex microbial floras in the human body, consisting of more than 700 different bacterial species [17]. *Streptococcus mutans* (*S. mutans*) are major cariogenic organisms. *S. mutans* coadhere or coaggregate with other microbial species, followed by proliferation and spreading into other sites in the oral mucosa modulated by the concerted action of genes and signaling molecules [18]. Conversely, denture-related stomatitis is a common condition wherein mild inflammation and redness of the oral mucous membrane occurs beneath a denture. In approximately 90% of the cases, Candida species are involved; they are normally a harmless component of the oral microbiota in many people [19]. Interestingly however, although it has been accepted for decades that *S. mutans* is the etiologic agent of dental caries, recent evidence indicates a high prevalence of *S. mutans* in dental biofilms where the fungal pathogen *Candida albicans* (*C. albicans*) resides, suggesting that the interaction between these diverse species may facilitate cariogenic development [18]. 

Furthermore, previous reports [15,16] described experiments comparing PMMAs implanted with F ions alone and PMMAs implanted with both F and Ag ions simultaneously, but they did not examine PMMAs implanted with Ag ions alone. Therefore, this paper examined the surface characteristics by XPS, contact angle and surface energy, and AFM analyses and the anti-microbial properties of PMMAs implanted with Ar, F, and Ag ions by PBII for oral microorganisms (*S. mutans* and *C. albican*s).

## 2. Materials and Methods 

### 2.1. Materials

PMMA (Clarex, Nitto Jushi Kogyo, Co., Ltd., Tokyo, Japan) plates having dimensions of 10 mm × 10 mm × 1 mm were used. The PMMA plates were modified by PBII equipment at Plasma Ion Assist Co., Ltd., Kyoto, Japan. The gases used for the ion implantation process were 100% Ar, or 95% Ar and 5% F_2_ (Ar/F_2_ gas). For the Ag ion implantation, a 99.8% Ag mesh cover was placed 10 mm above the PMMA plates and then sputtered by Ar or Ar/F_2_ gas at an acceleration voltage of –5 keV for 60 min. The experimental groups and the conditions of the treatments by PBII are listed in Table 1. Further, the Ar group was provided as a positive control group.

### 2.2. Elemental Analysis

The surface atomic changes of each specimen were characterized by XPS using an X-ray photoelectron spectrometer (PHI X-tool, ULVAC-PHI, Inc., Kanagawa, Japan) with an Al-Kα radiation source (15 kV 4 W, spot size: 19 µm). Peak decomposition was achieved using software (PHI MultiPak, ULVAC-PHI, Inc., Kanagawa, Japan), and the peak assignments were supported with Handbook of X-ray Photoelectron Spectroscopy (ULVAC-PHI, Inc., Kanagawa, Japan).

### 2.3. Contact Angle Measurement 

Specimens were ultrasonically cleaned in distilled water for 10 min and then dried in a clean bench at room temperature before the contact angle measurements were taken. Static contact angle measurements were conducted via the sessile drop technique using a contact angle meter (CA-DT, Kyowa Kaimenkagaku Co., Saitama, Japan) with three test liquids: distilled water, diiodomethane (CH_2_I_2_, 267.84 g/mol, >97.0%; FUJIFILM Wako Pure Chemical Co., Osaka, Japan), and ethylene glycol (C_2_H_6_O_2_, 62.07 g/mol, >99.5%; FUJIFILM Wako Pure Chemical Co., Osaka, Japan) at room temperature. One point per specimen was measured, and the number of specimens treated in the same manner was five (*n* = 5). The surface free energy of each sample group was calculated from the average of the contact angles of each test liquid, the known surface tension component values of each test solution [20] (shown in Table 2), the equations of Liu et al. [20] based on Young’s equation [21], and the Oss and Good acid-based method [22].

### 2.4. Atomic Force Microscopic Analysis

The surface morphologies of the specimens were characterized by atomic force microscopy (AFM: Nanosurf EasyScan2, Nanoscience Instruments Inc., Phoenix, AZ, USA) in the tapping mode in air. An AFM probe (BudgetSensor Tap190Al-G, Innovative Solutions Bulgaria Ltd., Sofia, Bulgaria) with a tip radius of <10 nm was used. The surface topography was observed, and the surface roughness was calculated. The evaluation of the roughness parameter of the sample was based on a scanned area of 378 nm^2^. 

### 2.5. Antimicrobial Test

Specimens were sterilized using a formalin gas sterilizer (Hollhope Dental ASK-30HP, ASUKAMEDICAL Co., Osaka, Japan) before the antimicrobial test. Two reference strains from the American Type Culture Collection, *S. mutans* (ATCC 25175) and *C. albicans* (ATCC 10261) were used in this study. An antimicrobial test was performed by the Adenosine-5’-triphosphate (ATP) luminescence method [16]. One loopful of each stock culture was transferred to a brain–heart infusion broth (BHI; Difco, Detroit, MI, USA) and incubated at 37 °C for 18 h. Cells of the resultant culture were washed twice with phosphate-buffered saline (PBS; FUJIFILM Wako Pure Chemical Co., Osaka, Japan) at 4000 rpm for 15 min and resuspended in a diluted BHI broth medium. The *S. mutans* and *C. albicans* cell suspensions were spectrophotometrically standardized to concentrations of 8 × 10^5^ and 3 × 10^5^ CFU/ml, respectively. The suspension (50 μl) was dropped to the bottom of a sterilized 24 well plate, covered by the PMMA specimen, and then incubated at 37 °C for 4 h. After incubation, PBS solution was poured into the vial and *S. mutans* or *C. albicans,* which adhered to PMMA specimens, were washed out by shaking. The ATP luminescence intensity of the suspension, which was expressed in relative luminescence units (RLU), was measured using an ATP-luciferase reaction kit (Lucifer HS, Kikkoman Co., Chiba, Japan) and a luminescence-measuring instrument (Lumitester C-110, Kikkoman Co., Chiba, Japan), according to the manufacturer’s instructions. Each specimen was measured three times. The number of specimens treated in the same manner was two (*n* = 5).

### 2.6. Statistical Analysis

Data were presented in the form of mean ± standard deviation (S.D.) and analyzed via one-way ANOVA and Tukey’s test (KaleidaGraph 4.00, SYNERGY SOFTWARE, Reading, PA, USA), with *p* < 0.05 indicating statistically significant results. The confidence interval was set at 95%.

## 3. Results

### 3.1. XPS Analysis

**Figure 1:**
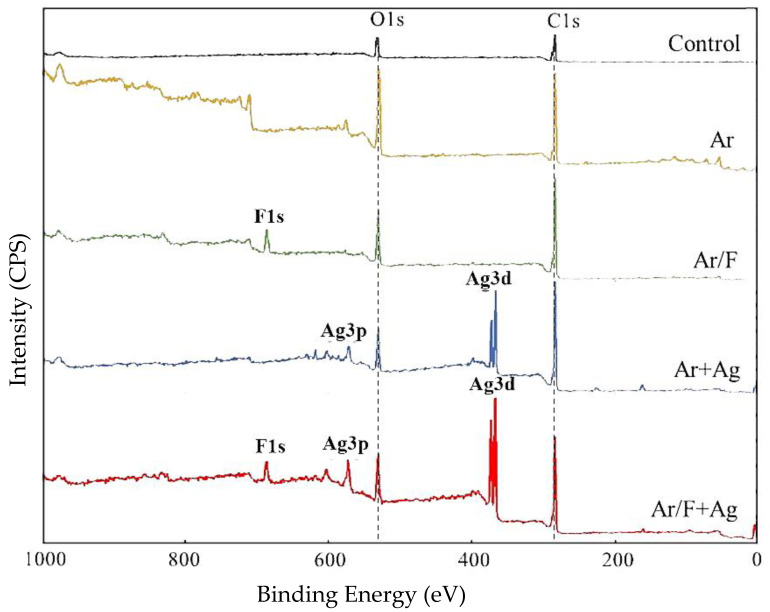
XPS wide-scan spectra from each PMMA group. Dashed lines indicate the peak positions of PMMA components. Bold characters indicate the peaks of implanted F and Ag.

**Figure 2:**
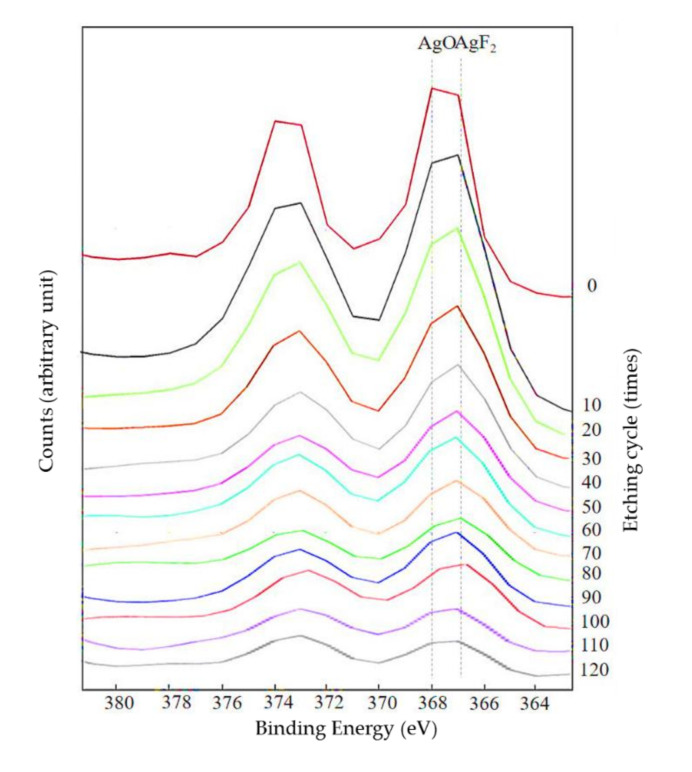
Ag3d depth profile spectra of Ar/F+Ag specimen. Separated peaks indicate AgO 368.0 eV and AgF_2_ 367.0 eV.

Figure 1 depicts the representative XPS wide-scan spectra from each group. The spectrum from the control surface of PMMA exhibited peaks at 286.0 and 534.0 eV, which may be assigned to C1s and O1s, respectively. The new peaks at approximately 689.0 eV in the spectrum from the Ar/F and Ar/F+Ag surface were characteristic of F1s. Additionally, another new peak was detected at approximately 369 eV in the spectrum from the Ar+Ag and Ar/F+Ag surface, which may be assigned to Ag3d. However, the Ar peak could not be confirmed. The Ag3d depth profile spectra of the Ar/F+Ag specimen are shown in Figure 2. At the most surface layer (at etching cycle = 0), the peak of AgO (368.0 eV) exceeded the peak of AgF_2_ (367.0 eV), in contrast, the peak of AgF_2_ was predominant internally. The Ag3d spectra on the surface were in the form of Ag oxide and exhibited AgF in the near-surface layer.

### 3.2. Contact Angle and Surface Energy Analysis

**Figure 3:**
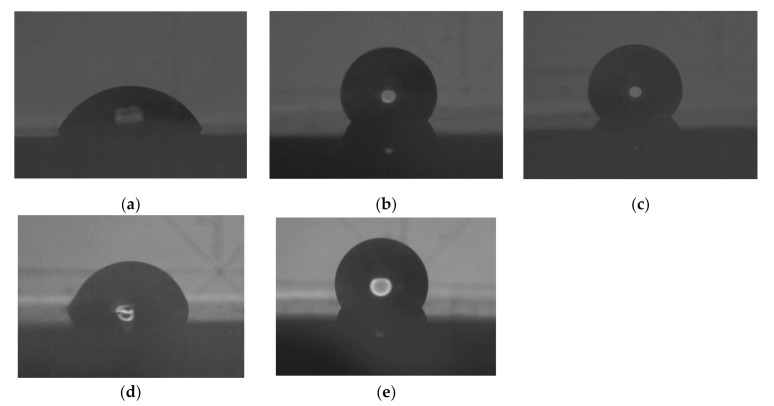
Images of the water drop on each PMMA specimen: (**a**) Control; (**b**) Ar; (**c**) Ar/F; (**d**) Ar+Ag; (**e**) Ar/F+Ag specimens.

**Figure 4:**
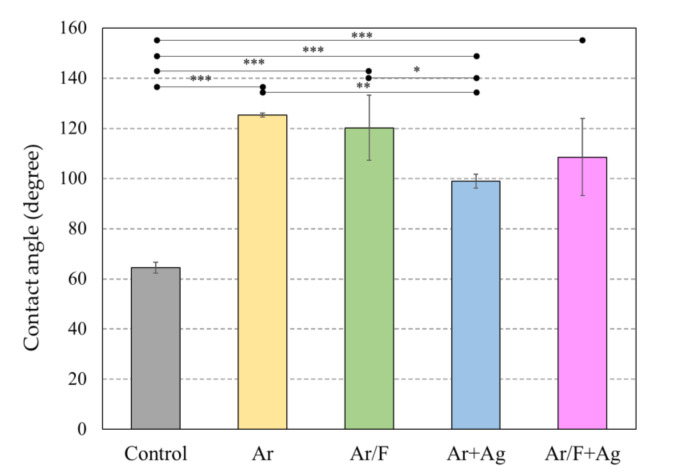
The water contact angles of each PMMA specimen. * *p* < 0.05, ** *p* < 0.01, *** *p* < 0.001 (ANOVA/Tukey, α = 0.05).

Figure 3 depicts images of the water drop, and Figure 4 depicts the water contact angles of each specimen. The values of the water contact angle were significantly higher for all ion-implanted groups than for the control PMMA. Comparison of the water contact angles between the ion-implanted groups, excluding the control group, shows that Ag implantation tended to reduce the water contact angle. In particular, the Ar+Ag group had the smallest water contact angle among all ion-implanted groups. It was clearly shown in Figure 3, however, there was a significant difference from the control group.

Additionally, Table 3 lists the contact angles of each specimen using water, diiodomethane, and ethylene glycol, and the values of the surface energy components calculated from the values of the contact angles. The Ar group had the largest surface free energy, and the other ion-implanted groups had an energy value similar to that of the control. 

### 3.3. AFM Analysis

**Figure 5:**
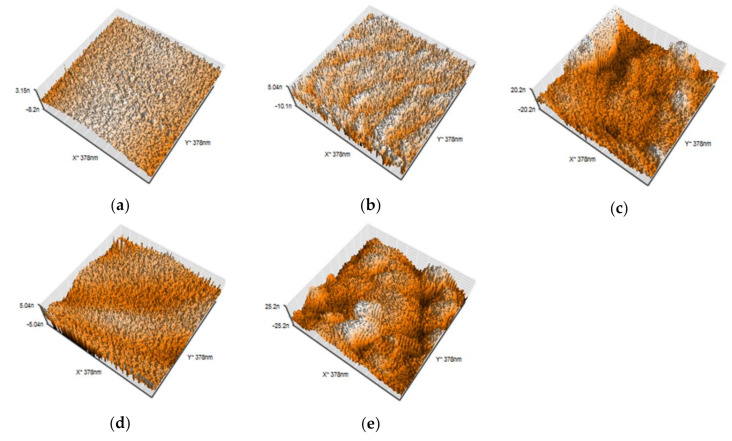
Three-dimensional AFM images: (**a**) Control; (**b**) Ar; (**c**) Ar/F; (**d**) Ar+Ag; (**e**) Ar/F+Ag specimens.

Figure 5 illustrates representative AFM topographic images of each specimen. Table 4 lists the arithmetical surface roughness profile (Ra) values. Compared with the control group, the surface roughness of the Ar/F group and the Ar/F+Ag group increased significantly, and there was no significant difference between the Ar group and the Ar+Ag group. Additionally, a significant increase in surface roughness was observed in the Ar/F group and the Ar/F+Ag group compared with those of the Ar group and the Ar+Ag group. AFM images exhibited a hillier structure in the Ar/F group and the Ar/F+Ag group as compared to the other groups.

### 3.4. Antibacterial Activity Against S. Mutans

**Figure 6:**
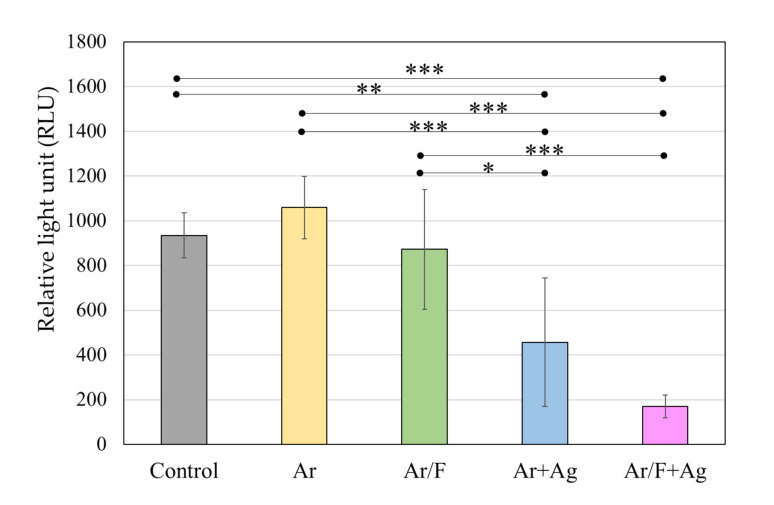
The luminescence intensity of the *S. mutans* suspension that was in contact with each specimen for four hours. * *p* < 0.05, ** *p* < 0.01, *p* < 0.001 (ANOVA/ Tukey, α = 0.05).

**Figure 7:**
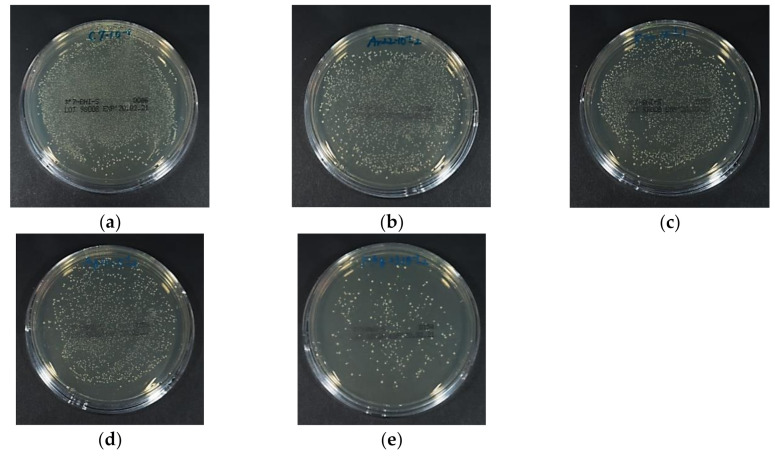
The representative images of the BHI agar medium in which the *S. mutans* suspension was spread and the colonies were grown. (**a**) Control; (**b**) Ar; (**c**) Ar/F; (**d**) Ar+Ag; (**e**) Ar/F+Ag specimens.

Figure 6 depicts the luminescence intensity of the *S. mutans* suspension that was in contact with each specimen for 4 h, and Figure 7 depicts representative images of the BHI agar medium in which the bacterial solution was spread and the colonies were grown. The ATP luminescence was measured and compared with that of the control group. Significant decreases in the amounts of ATP luminescence were observed in the Ar+Ag group and the Ar/F+Ag group. 

### 3.5. Antifungal Activity Against C. Albicans

**Figure 8:**
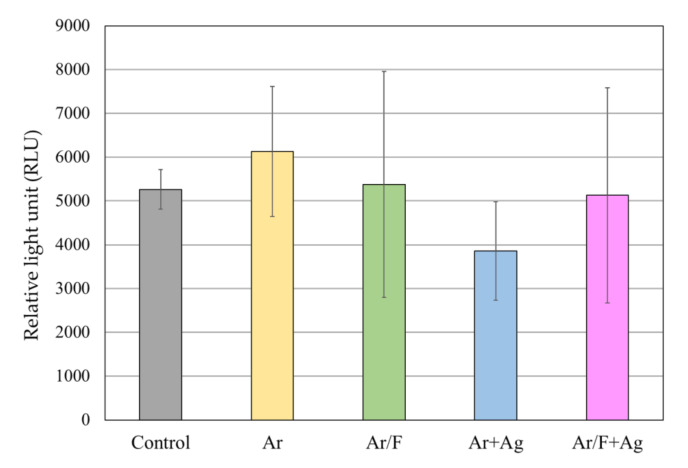
The luminescence intensity of the *C. albicans* suspension that was in contact with each specimen for four hours. There were no significant differences (ANOVA/ Tukey, α = 0.05).

**Figure 9:**
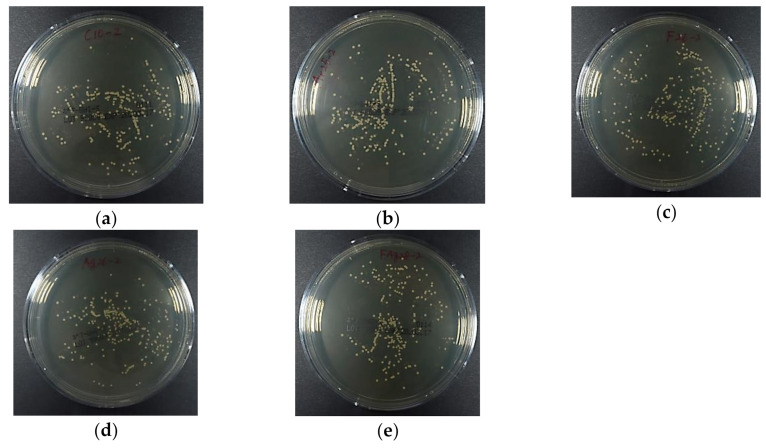
The representative images of the BHI agar medium in which the *C. albicans* suspension was spread and the colonies were grown: (**a**) Control; (**b**) Ar; (**c**) Ar/F; (**d**) Ar+Ag; (**e**) Ar/F+Ag specimens.

Figure 8 depicts the luminescence intensity of the *C. albicans* suspension after having been in contact with each specimen for 4 h. Figure 9 depicts the representative images of the BHI agar medium in which the bacterial solution was spread and the colonies were grown. The measured ATP luminescence indicated no significant difference in ATP luminescence between all groups. Images of the *C. albicans* colonies showed no apparent differences in colony numbers between all groups.

## 4. Discussion

In our previous study [15,16], C_3_F_8_ was used for F ion implantation to PMMA. However, C_3_F_8_ is a perfluorocarbon that was designated as a greenhouse substance by the Kyoto Protocol [23,24]. For environmental reasons, a mixed gas of 95% Ar (an inert gas) and 5% F_2_ (Ar/F gas) was used instead of C_3_F_8_ gas in the present study, and XPS analysis revealed that F and Ag ions could be implanted into PMMA using these gases. Moreover, previous studies [15,16] compared PMMAs implanted with F ions only or PMMAs simultaneously implanted with both F and Ag ions and did not examine PMMAs implanted with Ag ions only. The ultimate goal of this research is to provide surface modification by ion implantation to all intraoral devices to provide inhibition of bacterial adhesion. Previous studies have shown the effectiveness of F- and Ag-dual ion implantation in stainless steel and PMMA [15,16]. However, corrosion of titanium by anti-carious agents containing fluoride has been reported [25]. Additionally, it was observed that the surface of titanium plate became discolored and corroded after plasma-based fluorine ion implantation in our previous study [14]. Therefore, titanium can only be implanted with Ag; thus, in the present study, the effectiveness of only Ag ion implantation is additionally examined to determine the applicability of this method to various materials in the future. Considering such future prospects, the application of Ar in this method is very significant, because the hydrophobicity of the PMMA surface by Ar ion implantation was comparable to that by F ion implantation in this study. The improvement of the hydrophobicity of the surface of the materials, such as PMMA and titanium, by plasma treatment using Ar gas has been supported by several studies [26,27]. In this study, the peak of Ar implanted by PBII could not be found in the XPS analysis. However, the results of the water contact angle show that Ar was injected into the specimens from the contact angle and surface energy analysis (Figure 3 and Figure 4, and Table 3), and the AFM images (Figure 5). The antimicrobial properties on the Ar group are not presented in this study. However, Ar is useful for application to dental materials as the improvement of hydrophobicity affects the antifouling property. Additionally, reports show that plasma ion treatment using Ar gas promotes cell adhesion [28,29]. Therefore, in applying our method to titanium devices, such as orthodontic archwires and implants that could be corroded by the F ion, it was concluded that Ag ion implantation by Ar gas, which does not contain F ions, is the most effective due to its antibacterial effect and ability to promote cell attachment.

When comparing the effect of Ar gas and Ar/F gas on the surface properties and shape, the groups using Ar/F gas tended to have rougher surfaces from AFM observations and higher surface roughness values than the groups using Ar gas. The AFM images in Figure 5 show that the structural factor of the implanted F ions resulted in an increased surface roughness. Similarly, the result of the surface roughness in Table 4 suggests that F ion implantation increased surface roughness. However, there was no difference in antimicrobial activity due to the surface morphological differences. It has been highlighted that rougher surfaces possess a greater surface area and the depressions in the roughened surfaces can provide more favorable sites for colonization [30]. Bollen et al. [31] reported that bacterial adhesion is primarily determined by a surface roughness of Ra > 200 nm. Additionally, Lee et al. [32] reported that the total amount of bacteria adherent on resin (Ra = 179 nm) was significantly higher than that on titanium and zirconia, which had lower Ra values of 59 and 64 nm, respectively. In this study, the smallest surface roughness was 1.01 nm on the Ar+Ag group, and the greatest was 4.94 nm on the Ar/F+Ag group. This difference was insufficient to effect change in the morphology and affect bacterial adhesion.

The antimicrobial effects of Ag occur by the following mechanism: (1) destruction of oxidation catalyzed by Ag, (2) disruption of electron transfer in the bacteria by monovalent Ag, and/or prevention of the unwinding of DNA in the viruses with the substitution of H ions by monovalent Ag, and (3) destruction of bacteria and viruses by bivalent and trivalent Ag [33]. Therefore, Ag has been used in different medical and dental fields for centuries. Additional advantages of Ag include low toxicity, good biocompatibility with human cells, long-term antibacterial activity due to sustained ion release, and low bacterial resistance [34]. Our previous study [15] confirmed that both Ag- and F-ion implanted PMMA had antibacterial activity from *S. aureus*. Additionally, experiments in this study demonstrated that Ag ion and F/Ag dual ion implantation by PBII induced an antibacterial effect against *S. mutans* on the PMMA surface. However, they did not affect *C. albicans*. More recently, silver nanoparticles (AgNP) have been incorporated into several biomaterials, and many researchers have reported their effects against bacteria and fungus [34]. Nam [35] incorporated AgNPs into a commercial tissue conditioner at concentrations of 0.1% to 3.0% and evaluated its antimicrobial effects against *S. aureus*, *S. mutans*, and *C. albicans*. The author reported that the susceptibility of *C. albicans* to AgNP-tissue conditioner sample (0.5%) was less than that of the samples (0.1%) of *S. aureus* and *S. mutans*. Moreover, Lu [36] demonstrated that eukaryotes, such as fungus, could use multiple cellular strategies to cope with AgNPs stress, whereas bacteria were negatively affected by AgNPs. These findings suggest that the differences in the antimicrobial properties between *S. mutans* and *C. albicans* in the present study were dependent on the Ag concentration on the surface of our PMMA specimens and the susceptibility of *C. albicans* to Ag. Moreover, Panáček [37] found that ionic Ag inhibited the growth of the tested yeasts at concentrations comparable to the cytotoxic level (approximately 1 mg/L) of ionic Ag against the tested human fibroblasts. This finding suggests that a concentration of Ag that exerts an antifungal effect against *C. albicans* may cause cytotoxicity in the human body.

In this study, there was no antibacterial activity against *S. mutans* in the Ar/F group implanted with F ions alone. Several studies have reported the use of F alone or a combination of F and Ag for dental materials to obtain an anti-cariogenic effect [38,39]. Lou et al. [38] evaluated the antimicrobial effects of Ag and F agents against *S. mutans*, *Lactobacillus acidophilus*, and *Actinomyces naeslundii* and identified the components that are effective against these bacteria. The conclusion was that Ag ions appear to be the principal antibacterial agent at both high and low concentrations, whereas F ions only have antibacterial effects at high concentrations. In another study [39], an experimental dentifrice containing nano-silver fluoride (NSF) with AgNP and a sodium fluoride (NaF) toothpaste were tested in vitro to evaluate the antibacterial property against *S. mutans*. It concluded that NSF, which includes both Ag and F, had a better antibacterial effect than NaF, Ag excluded. These findings indicated that only Ag ions appear to possess an anti-caries effect under the conditions of low concentrations of F and Ag ions. Therefore, it may be said that only Ag ions should be implanted to PMMA for an antibacterial activity. Regarding the antibacterial activity against *S. mutans*, no significant difference was observed between the Ar+Ag group and the Ar/F+Ag group; however, the Ar/F+Ag group tended to suppress the growth of *S. mutans* more than the Ar+Ag group. Tsuji et al. [40] indicated that Ag-negative-ion implantation was found to lower the contact angle of a polystyrene surface with an ion energy below 20 keV. In this study, the water contact angle between the groups treated with the same gas (Ar vs Ar+Ag, or Ar/F vs Ar/F+Ag), and the Ag implanted groups (Ar+Ag and Ar/F+Ag) tended to reduce hydrophobicity. It is well-known that F enhances the hydrophobicity of solid surfaces [41,42]. Tang et al. concluded that there were fewer adhered bacteria on the hydrophobic surface, and they did not readily clump together [43]. Yoda et al. reported that Co-Cr-Mo, which has a more hydrophobic surface, demonstrated a lower bacterial adherence than other materials [30]. Based on these findings, it is considered that the Ar/F+Ag group showed the highest antibacterial activity against *S. mutans* due to a synergistic effect, which is the antibacterial effect of the Ag ions and the hydrophobicity suppressing bacterial adhesion by the F ions.

However, the antimicrobial test in this study has its limitations. For instance, the incubation time was set to a reduced time of 4 h to control the evaporation of the bacterial solution, because a very small amount of the bacterial solution was brought into contact with the sample surface. This was so to minimize the amount of bacterial solution in contact with only one ion-implanted surface. It was considered that the antibacterial properties of Ag ion on the sample surface were exhibited before *S. mutans* was stacked on the sample surface because the incubation was performed in a short time using a small amount of bacterial solution. Whether the antibacterial effect of Ag ions penetrated multiple bacterial layers that adhered to the sample surface by increasing the incubation time or increasing the amount of bacterial solution in contact with the sample was beyond the scope of this work and should be examined in future studies.

## 5. Conclusions

In the present study, PMMA plates were simultaneously implanted with both F and Ag ions using Ar or Ar/F gases and Ag mesh by a PBII process. The following results were obtained. (1) Fluorine and silver ions were implanted in PMMA via PBII with Ar gas. (2) Surface contact angle was significantly increased by both ion implantations. (3) Surface roughness changed due to these ions implantations by less than 5 nanometers. (4) Fluorine and silver ions implantations inhibited the growth of *S. mutans*, but no significant antifungal activity against *C. albicans*. 

Ar is an environmentally friendly gas; thus, it had no significant physical or chemical effects on our modification method, which made Ar gas suitable for the practical application of our method. F ion implantation improved the hydrophobicity of the surface of the PMMA sample which can reduce bacterial attachment and aggregation, and the antibacterial effect of the Ag ions gave the PMMA sample antibacterial properties against oral bacteria. However, no antifungal effect was obtained.

## Figures and Tables

**Table 1:** Experimental groups.

Group	Gas	Ag Mesh
Control	-	-
Ar	100% Ar	-
Ar/F	95% Ar, 5% F_2_	-
Ar+Ag	100% Ar	+
Ar/F+Ag	95% Ar, 5% F_2_	+

**Table 2:** Test liquids and their surface tension components *.

Surface Tension Data (mJ/m^2^)	*γ*_L_	*γ*_L_^LW^	*γ*_L_^AB^	*γ*_L_^+^	*γ*_L_^−^
Water (W), H_2_O	72.8	21.8	51.0	25.5	25.5
Diiodomethane (D), CH_2_I_2_	50.8	50.8	0	0	0
Ethylene Glycol (E), C_2_H_6_O_2_	48.0	29.0	19.0	1.92	47.0.

* *γ*_L_: surface tension of the liquid, *γ*_L_^LW^: Lifshitz-van der Waals apolar component of the liquid, *γ*_L_^AB^: Lewis acid-base polar component of the liquid, *γ*_L_^+^: electron acceptor subcomponent of the liquid, *γ*_L_^−^: electron donor subcomponent of the liquid.

**Table 3:** Contact angles and Surface free energy *.

Group	Contact Angle θ (Degree)			Surface Energy Components (mJ/m^2^)				
	*θ* ^W^	*θ* ^Di^	*θ* ^EG^	*γ* ^LW^	*γ* ^+^	*γ* ^−^	*γ* ^AB^	*γ* ^TOT^
Control	64.4	36.3	50.8	41.42	0.08	21.67	2.63	44.05
Ar	125.3	32.6	42.2	43.11	3.77	24.69	19.29	62.41
Ar/F	120.2	54.4	68.7	31.79	0.78	6.32	4.44	36.23
Ar+Ag	99.0	43.8	62.3	37.65	0.08	0.02	0.07	37.72
Ar/F+Ag	108.5	43.3	62.6	37.91	0.77	8.94	5.25	43.16

* θ^W^: contact angle of distilled water, *θ*^Di^: contact angle of diiodomethane, *θ*^EG^: contact angle of ethylene glycol, *γ*^LW^: Lifshitz-van der Waals apolar component, *γ*^+^: electron acceptor subcomponent, *γ*^−^: electron donor subcomponent, *γ*^AB^: Lewis acid-base polar component, *γ*^TOT^: the total surface free energy.

**Table 4:** Surface roughness.

Group	Mean ± S.D (nm)		Maximum (nm)	Minimum (nm)
Control	1.66 ± 1.17	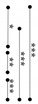	3.18	0.30
Ar	1.69 ± 0.19		1.92	1.49
Ar/F	3.54 ± 1.78		5.64	1.61
Ar+Ag	1.01 ± 0.04		1.04	0.95
Ar/F+Ag	4.94 ± 0.60		5.65	4.23

* *p* < 0.05, ** *p* < 0.01, *** *p* < 0.001 (ANOVA/ Tukey, α = 0.05)
